# Comparative Longitudinal Evaluation of Systemic Inflammatory Markers in Type 2 Diabetes Treated with Four Oral Antidiabetic Drug Classes

**DOI:** 10.3390/jcm15020688

**Published:** 2026-01-15

**Authors:** Mehmet Yamak, Serkan Çakır, Sami Uzun, Egemen Cebeci, Özlem Menken, Savas Ozturk

**Affiliations:** 1 Department of Internal Medicine, University of Health Sciences Haseki Training and Research Hospital, 34096 Istanbul, Turkey; 2 Department of Internal Medicine, Bayburt State Hospital, 69000 Bayburt, Turkey; serkancakir724@hotmail.com; 3 Department of Nephrology Medicine, University of Health Sciences Haseki Training and Research Hospital, 34096 Istanbul, Turkey; drsamiuzun@gmail.com (S.U.); dregemencebeci@hotmail.com (E.C.); 4 Department of Internal Medicine, Defne State Hospital, 31160 Hatay, Turkey; ozlemmenken@gmail.com; 5 Department of Nephrology, Istanbul Faculty of Medicine, Istanbul University, 34104 Istanbul, Turkey; savasozturkdr@yahoo.com

**Keywords:** type 2 diabetes mellitus, systemic inflammation, Systemic Immune-Inflammation Index, SGLT-2 inhibitors, DPP-4 inhibitors, Thiazolidinediones, hematologic markers, longitudinal analysis

## Abstract

**Background:** Systemic inflammation plays a central role in the pathogenesis and progression of type 2 diabetes mellitus (T2DM). Hematologic inflammatory indices-such as the Systemic Immune-Inflammation Index (SII), Neutrophil-to-Lymphocyte Ratio (NLR), Platelet-to-Lymphocyte Ratio (PLR), and Monocyte-to-Lymphocyte Ratio (MLR)-have emerged as accessible markers of chronic inflammation, yet longitudinal comparisons across oral antidiabetic therapies remain limited. This study uniquely integrates longitudinal correlation and network analyses in a large real-world T2DM cohort, allowing assessment of the temporal stability and class-specific inflammatory patterns across four oral antidiabetic therapies. **Methods:** This retrospective, longitudinal study analyzed 13,425 patients with T2DM treated with Biguanidines, Dipeptidyl Peptidase-4 (DPP-4) inhibitors, Sodium–Glucose Cotransporter-2 (SGLT-2) inhibitors or Thiazolidinediones (TZDs) between 2020 and 2024. Data were retrieved from the Probel^®^ Hospital Information System and included baseline, early (30–180 days), and late (180–360 days) follow-up laboratory results. Systemic inflammatory indices were computed from hematologic parameters, and correlations among inflammatory and biochemical markers were assessed using Spearman’s coefficients. **Results:** At baseline, all hematologic indices were strongly intercorrelated (SII–NLR r = 0.83, *p* < 0.001; SII–PLR r = 0.73, *p* < 0.001), with moderate associations to C-reactive protein (CRP; r ≈ 0.3–0.4) and weak or no correlations with Ferritin (r ≈ −0.1). These relationships remained stable throughout follow-up, confirming reproducibility of systemic inflammatory coupling. Longitudinally, SII and NLR showed modest early increases followed by significant declines at one year (*p* < 0.05), while PLR and MLR remained stable. Class-specific differences were observed: SGLT-2 inhibitors and TZDs demonstrated stronger and more integrated anti-inflammatory networks, whereas Biguanidines and DPP-4 inhibitors exhibited moderate coherence. Principal Component Analysis (PCA) explained 62.4% of total variance and revealed distinct clustering for TZD and SGLT-2 groups, reflecting class-specific inflammatory modulation. **Conclusions:** Systemic inflammatory indices (SII, NLR, PLR) provide reproducible and accessible measures of low-grade inflammation in T2DM. Despite overall inflammation reduction with treatment, drug-specific patterns emerged-SGLT-2 inhibitors and TZDs showed greater anti-inflammatory coherence, while Biguanidines and DPP-4 inhibitors maintained moderate effects.

## 1. Introduction

Type 2 diabetes mellitus (T2DM) is recognized not only as a metabolic disorder characterized by chronic hyperglycemia but also as a condition associated with persistent low-grade systemic inflammation, which contributes to the development of micro- and macrovascular complications. Chronic inflammatory processes in T2DM are reflected by elevated circulating biomarkers such as C-reactive protein (CRP) and leukocyte-derived indices, which correlate with disease progression and vascular risk [1,2]. Complete blood count-derived indices, including the neutrophil-to-lymphocyte ratio (NLR), platelet-to-lymphocyte ratio (PLR), and the Systemic Immune-Inflammation Index (SII), integrate innate and adaptive immune responses and have emerged as accessible markers of systemic inflammation in T2DM and its complications [3]. These markers have shown associations with diabetic nephropathy, retinopathy, and increased risk of adverse cardiovascular outcomes, demonstrating their clinical relevance beyond traditional biochemical measures such as CRP [4]. In addition to their diagnostic and prognostic value, systemic inflammatory markers are increasingly used to monitor treatment responses in metabolic disease. Certain antidiabetic drug classes, notably sodium-glucose cotransporter-2 (SGLT-2) inhibitors, exhibit anti-inflammatory and immunomodulatory effects that may be independent of glycemic control, including suppression of pro-inflammatory cytokines and macrophage activation pathways. Thiazolidinediones (TZDs) also modulate inflammation through peroxisome proliferator-activated receptor-γ (PPAR-γ)-mediated effects on adipose tissue and immune cell function, while dipeptidyl peptidase-4 (DPP-4) inhibitors have more modest immunomodulatory profiles. Despite growing evidence, longitudinal comparisons of systemic inflammatory indices across major oral antidiabetic therapies remain limited, warranting comprehensive evaluation of temporal inflammatory dynamics under real-world treatment conditions.

Unlike previous cross-sectional or short-term studies, the present work provides a comprehensive longitudinal comparison of systemic inflammatory indices among four oral antidiabetic drug classes, employing network and PCA-based approaches to characterize class-specific anti-inflammatory coherence and long-term correlation stability.

The primary objective of this study was to perform a comparative longitudinal analysis of systemic inflammatory indices-including the Systemic Immune-Inflammation SII, NLR, PLR, and Monocyte-to-Lymphocyte Ratio (MLR)-among patients with T2DM treated with four major oral antidiabetic drug classes under real-world conditions. The secondary objectives were (1) to examine the temporal evolution and stability of hematologic-biochemical inflammatory relationships (SII-NLR-PLR-CRP-Ferritin coupling) across one year, and (2) to identify drug class–specific patterns of systemic inflammatory modulation.

## 2. Materials and Methods

### 2.1. Study Design and Data Source

This retrospective, longitudinal study was conducted in the internal medicine outpatient clinics of our hospital, a tertiary referral center in the city, between January 2020 and May 2024. All patient information was obtained from the hospital’s Probel^®^ Hospital Information System, which centrally stores electronic medical records, prescriptions, and laboratory test results. The study protocol was approved by the institutional ethics committee (Approval No: 21-2024) and conducted in accordance with the Declaration of Helsinki. Data extraction from the Probel^®^ system included demographic information (age, sex, nationality), prescription details, and laboratory results.

### 2.2. Study Population and Follow-Up Periods

Patients aged between 18 and 75 years with a confirmed diagnosis of Type 2 Diabetes Mellitus (T2DM) were included in this study. Eligible participants were those who were initiated for the first time on one of the four oral antidiabetic drug classes—Biguanidines (Metformin), Dipeptidyl Peptidase-4 (DPP-4) inhibitors, Sodium-Glucose Cotransporter-2 (SGLT-2) inhibitors, or Thiazolidinediones (TZDs)—and who had complete laboratory data available for all three timepoints: baseline (before treatment initiation), early follow-up (30–180 days), and late follow-up (180–360 days). Patients were excluded if their records contained duplicates in the Probel^®^ hospital database (*n* = 4596), if baseline SII (Systemic Immune-Inflammation Index) values were missing (*n* = 432), or if data were incomplete for either the early or late follow-up period (*n* = 35). After data cleaning and exclusion of ineligible records, only patients with complete datasets across all three timepoints were retained for the final analysis.

### 2.3. Data Cleaning and Processing

The retrieved data were exported to Microsoft Excel (.xlsx) format and further processed for statistical analysis. These data underwent multistage cleaning using Python (Pandas, NumPy) and R. From hematologic parameters, the following inflammatory indices were derived at each timepoint:

SII (Systemic Immune-Inflammation Index) = (Neutrophils × Platelets)/Lymphocytes

NLR (Neutrophil-to-Lymphocyte Ratio) = Neutrophils/Lymphocytes

PLR (Platelet-to-Lymphocyte Ratio) = Platelets/Lymphocytes

MLR (Monocyte-to-Lymphocyte Ratio) = Monocytes/Lymphocytes

In addition, CRP, WBC, and Ferritin were analyzed as direct inflammatory markers.

### 2.4. Statistical Analysis and Visualization

All analyses were performed using a combination of SPSS Statistics v26.0 (IBM Corp.), R v4.3.1, and Python v3.10 within the Google Colab environment. Continuous variables were tested for normality with the Shapiro–Wilk test and expressed as median (IQR). Between-group comparisons were made using the Kruskal–Wallis test with Dunn-Bonferroni post hoc correction, and within-group comparisons with the Wilcoxon signed-rank test. Categorical variables were compared using the Chi-square or Fisher’s exact test. Spearman’s correlation coefficients (ρ) were calculated between inflammatory indices for each drug group and timepoint. Significant correlations (|ρ| ≥ 0.25, *p* < 0.01) were visualized through heatmaps and network graphs, where edge width represented correlation strength. Longitudinal correlation dynamics were depicted as temporal network evolution graphs. Principal Component Analysis (PCA) using scikit-learn was applied to evaluate clustering and shared inflammatory profiles among drug classes. Figures and tables were generated using Matplotlib (version 3.10.8), Seaborn (version 0.13.2), and NetworkX (version 3.6.1) in Python.

## 3. Results

**Baseline Characteristics:** A total of 13,425 patients with type 2 diabetes mellitus were analyzed. The mean age was 56.1 ± 10.6 years, with 62.3% female and 97.5% of Turkish nationality. Patients were distributed into four oral antidiabetic treatment groups: Biguanidines (*n* = 6033), DPP-4 inhibitors (*n* = 4675), SGLT-2 inhibitors (*n* = 1510), and Thiazolidinediones (*n* = 1207). As shown in Table 1, the DPP-4 and SGLT-2 groups were significantly older and had higher baseline HbA1c and glucose levels than the Biguanidine and Thiazolidinedione groups (*p* < 0.001). Gender distribution also differed significantly, with more males in the DPP-4 and SGLT-2 groups (*p* < 0.001). Inflammatory indices such as SII and NLR were elevated in the DPP-4 and SGLT-2 groups (*p* < 0.05). Overall, patients in the DPP-4 and SGLT-2 groups exhibited higher baseline systemic inflammation and poorer glycemic control compared with those in the Biguanidine or TZD groups.

**Temporal Changes in Glycemic and Inflammatory Markers:** Changes in glycemic and inflammatory parameters over time are summarized in Table 2. At both 30–180 and 180–360 days, HbA1c and glucose decreased significantly across all groups, with the greatest improvement observed in Biguanidine users. Similarly, systemic inflammatory markers including SII and NLR declined over time, indicating partial resolution of inflammation during treatment. CRP levels showed modest but significant reductions, while Ferritin and PLR changes were minimal. Overall, the reduction in inflammatory indices paralleled metabolic improvement, particularly among Biguanidine and TZD users. Given the large sample size, small numerical differences often reached statistical significance; however, these should be interpreted in light of their modest absolute effect sizes.

**Correlation Analyses of Inflammatory Parameters (Baseline):** As shown in Table 3, SII was strongly and positively correlated with NLR and PLR in all groups, confirming the strong internal coherence of hematologic inflammatory indices. Moderate positive correlations were observed between SII and WBC and between SII and CRP, whereas Ferritin displayed weak or no correlation with SII. Among drug classes, DPP-4 and SGLT-2 inhibitor users exhibited slightly higher SII-CRP correlations compared to Biguanidine and TZD users.

**Correlation Analyses at 30–180 Days (Follow-up 1):** Correlation coefficients among inflammatory parameters at the first follow-up are summarized in Table 4. All major associations between inflammatory markers persisted after 3–6 months of treatment. The correlations between SII_1_ and NLR_1_ remained strong (r = 0.81–0.84, *p* < 0.001) across all groups, indicating preserved interdependence between composite inflammatory indices. Similarly, SII_1_ and PLR_1_ correlations remained robust, while moderate correlations were maintained between SII_1_ and CRP_1_ and SII_1_ and WBC_1_. Ferritin_1_ remained weakly or negatively correlated with inflammatory indices. Among the treatment groups, DPP-4 and SGLT-2 inhibitor users continued to show the strongest SII-NLR_1_ associations, suggesting sustained inflammatory coupling despite glycemic improvements.

**Figure 1:**
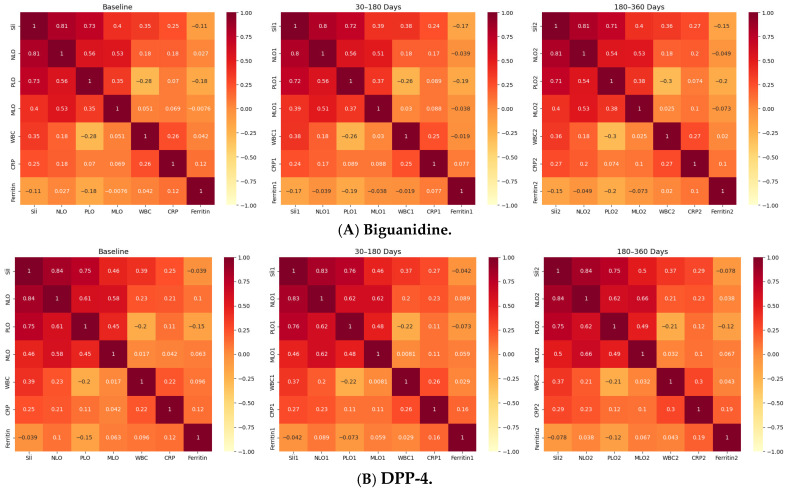

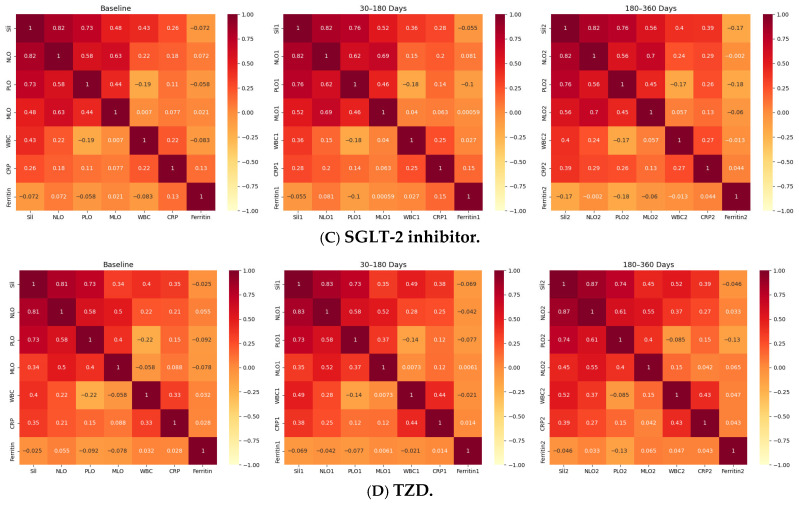
Longitudinal Correlation Heatmaps of Inflammatory Indices Across Drug Classes. Figure depicts the correlations among systemic inflammatory indices-Systemic Immune-Inflammation Index (SII), Neutrophil-to-Lymphocyte Ratio (NLR), Platelet-to-Lymphocyte Ratio (PLR), and Monocyte-to-Lymphocyte Ratio (MLR)-alongside WBC, C-reactive protein (CRP), and Ferritin, across three timepoints (baseline, 30–180 days, 180–360 days) within the four oral antidiabetic groups (**A**): Biguanidine, (**B**): DPP-4 inhibitors, (**C**): SGLT-2 inhibitors, (**D**): Thiazolidinediones. Each heatmap shows Spearman’s correlation coefficients (ρ), with red indicating strong positive (ρ > 0.7), yellow moderate (ρ = 0.3–0.7), and pale tones weak or negative (ρ < 0.3) associations. Across all treatments, SII-NLR correlations remained very strong (ρ = 0.81–0.84, *p* < 0.001), confirming the internal consistency of hematologic inflammatory markers. SII-PLR and NLR-PLR correlations were moderately strong (ρ ≈ 0.48–0.75), while SII-CRP and NLR-CRP correlations were weaker but significant (ρ = 0.28–0.37, *p* < 0.001). Ferritin showed weak or inverse relationships (ρ ≈ −0.05 to −0.14) in all groups. The DPP-4 and SGLT-2 inhibitor groups displayed the most coherent inflammatory patterns, with consistent correlation matrices across all timepoints, whereas Biguanidine and TZD users exhibited milder or less interconnected profiles. Overall, these results demonstrate that systemic inflammatory indices in type 2 diabetes maintain a stable correlation structure over time, with drug-specific variations in network strength and coherence.

**Figure 2:**
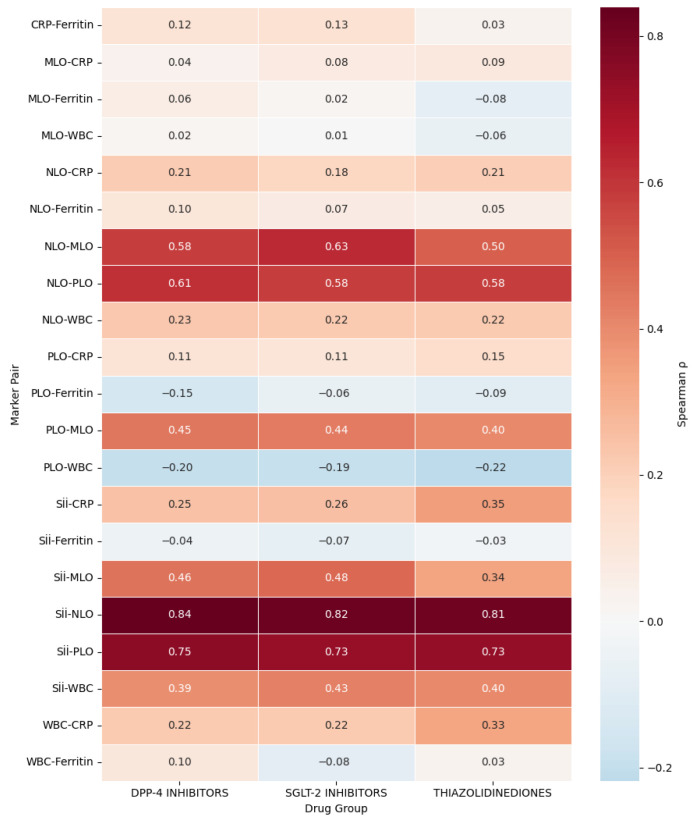
Comparative Correlation Heatmap of Inflammatory Markers Across Oral Antidiabetic Drug Classes (Biguanidine is the Reference Group). Spearman correlation coefficients (ρ) between systemic inflammatory indices are displayed for Dipeptidyl Peptidase-4 (DPP-4) inhibitors, Sodium-Glucose Cotransporter-2 (SGLT-2) inhibitors, and Thiazolidinediones (TZDs). The Biguanidine group was excluded from this figure as it served as the reference cohort in comparative analyses, representing the baseline metabolic and inflammatory profile. Red shades indicate strong positive correlations (ρ > 0.6), orange moderate correlations (ρ = 0.3–0.6), and blue weak or negative associations (ρ < 0). Across all classes, the strongest correlations were observed between the Systemic Immune-Inflammation Index (SII) and the Neutrophil-to-Lymphocyte Ratio (NLR) (ρ ≈ 0.83), followed by SII-Platelet-to-Lymphocyte Ratio (PLR) (ρ ≈ 0.74). Moderate correlations were found between SII and C-Reactive Protein (CRP) (ρ ≈ 0.30–0.40) and between SII and White Blood Cell count (WBC) (ρ ≈ 0.39–0.43), whereas Ferritin showed minimal or negative associations (ρ ≈ −0.15 to +0.13). The TZD group exhibited slightly higher SII-CRP (ρ = 0.35) and WBC-CRP (ρ = 0.33) correlations, suggesting stronger hematologic-biochemical coupling compared with DPP-4 and SGLT-2 groups.

**Correlation Analyses at 180–360 Days (Follow-up 2):** At one-year follow-up (Table 5), correlations among inflammatory markers remained strong and consistent across all drug classes (Figure 1). The SII_2_-NLR_2_ relationship was very strong (r = 0.83–0.84, *p* < 0.001), confirming the long-term stability of systemic inflammatory coupling. SII_2_-PLR_2_ correlations also remained high, while SII_2_-CRP_2_ associations slightly increased compared with earlier follow-ups. The SGLT-2 inhibitor group showed the highest SII-CRP correlation. Ferritin_2_ correlations were weak or nonsignificant in all groups. Comparative mapping across classes (Figure 2) showed similar results: SII-NLR remained the strongest link, followed by SII-PLR, SII-MLR, and NLR-PLR. Correlations involving biochemical markers were moderate to weak-SII-CRP, SII-WBC and Ferritin. Among therapies, Thiazolidinediones showed the most integrated profile, with higher SII-CRP and WBC-CRP relationships, while DPP-4 and SGLT-2 inhibitors demonstrated moderate internal coherence. Biguanidines displayed a simpler network structure. Overall, the SII-NLR-PLR axis remained the most stable inflammatory core in type 2 diabetes, whereas CRP and Ferritin exhibited variable, drug class-dependent associations.

**Figure 3:**
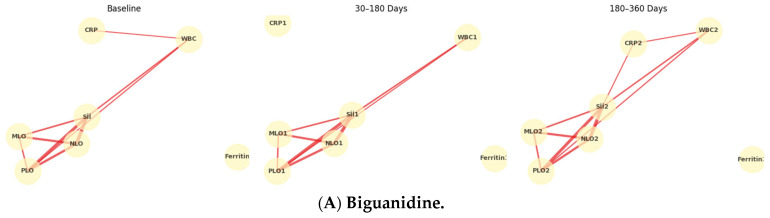

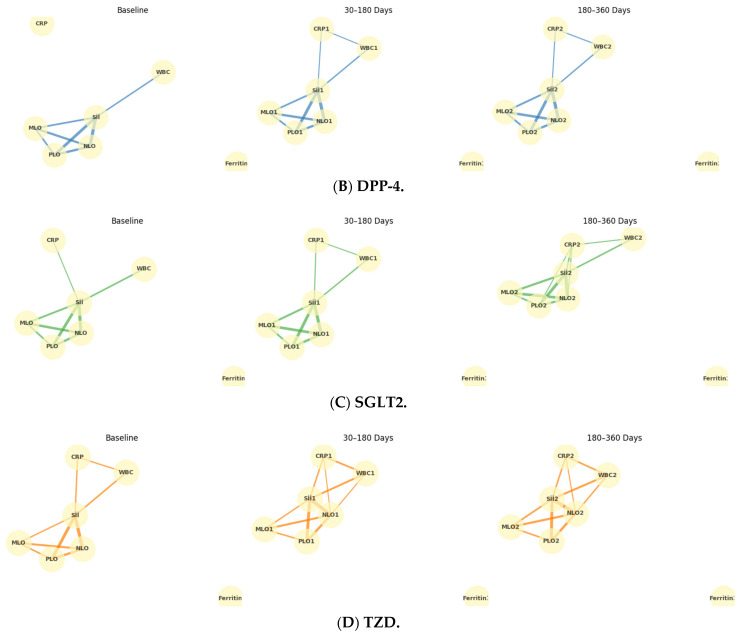
**Temporal evolution of inflammatory correlation networks across antidiabetic drug classes.** Panels show (**A**) Biguanidine, (**B**) DPP-4 inhibitors, (**C**) SGLT-2 inhibitors, and (**D**) Thiazolidinediones at three follow-up intervals (Baseline, 30–180 days, 180–360 days). Nodes represent systemic inflammatory indices (SII, NLR, PLR, MLR), leukocyte count (WBC), C-reactive protein (CRP), and Ferritin. Edges denote Spearman’s ρ correlations (|ρ| ≥ 0.25), with line thickness proportional to correlation strength. Each drug class shows a persistent central SII-NLR-PLR-MLR cluster, while peripheral nodes (CRP, WBC, Ferritin) display time-dependent reorganization, indicating distinct inflammatory modulation trajectories under different therapeutic mechanisms.

**Figure 4:**
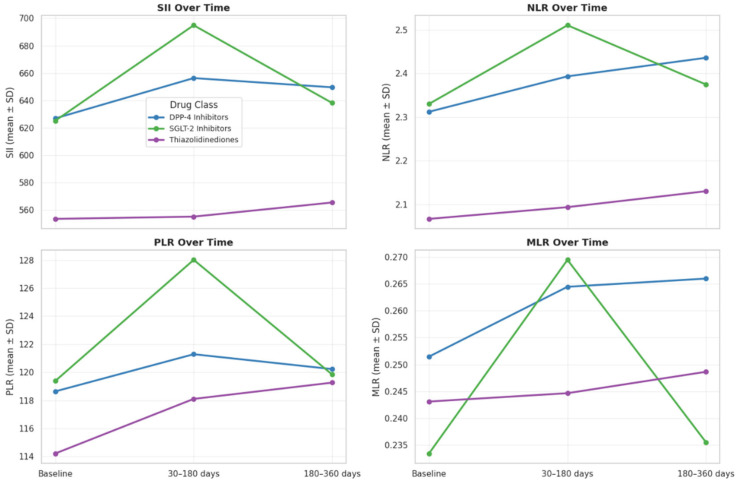
Temporal changes in systemic inflammatory indices (SII, NLR, PLR, MLR) at baseline, 30–180 days, and 180–360 days by oral antidiabetic drug class. Each line shows group mean ± SD. SII and NLR slightly increased early and declined at one year, while PLR and MLR remained stable. The TZD group showed the lowest, and the SGLT-2 group the highest early inflammation levels.

**Temporal Evolution of Inflammatory Networks Across Drug Classes:** The temporal network analysis revealed distinct yet overlapping inflammatory correlation patterns among the four drug classes (Figure 3A–D). Across all treatments, a stable hematologic core (SII-NLR-PLR-MLR) persisted throughout 12 months, indicating durable systemic inflammatory coupling. In the Biguanidine group (Figure 3A), the baseline network showed a dense SII-NLR-PLR cluster with moderate WBC and CRP connections. At 30–180 days, CRP and Ferritin links weakened, suggesting reduced acute-phase activity, while partial SII-CRP and SII-WBC reconnections emerged by 360 days. The DPP-4 inhibitor network (Figure 3B) maintained strong, widespread correlations at all timepoints, with CRP and WBC consistently integrated into the core. In the SGLT-2 inhibitor group (Figure 3C), the network became progressively compact and symmetric, with reinforced SII-NLR-PLR-MLR links and reappearance of CRP and WBC associations at one year. The Thiazolidinedione group (Figure 3D) showed the broadest connectivity. From baseline to 180 days, inter-variable edges strengthened across all markers, and at 12 months, a dense, multi-nodal configuration persisted with CRP and WBC embedded centrally, reflecting sustained systemic integration. Figure 4 illustrates the longitudinal evolution of the systemic inflammatory indices—Systemic Immune-Inflammation Index (SII), Neutrophil-to-Lymphocyte Ratio (NLR), Platelet-to-Lymphocyte Ratio (PLR), and Monocyte-to-Lymphocyte Ratio (MLR)—across baseline, early follow-up (30–180 days), and late follow-up (180–360 days) periods in patients receiving different oral antidiabetic treatments. Across all drug groups, SII and NLR showed mild early increases followed by gradual decreases at one year, while PLR and MLR remained relatively stable. The Thiazolidinedione group maintained the lowest overall inflammatory indices throughout follow-up, whereas SGLT-2 inhibitors exhibited the highest early SII and NLR peaks, which declined toward late follow-up. The Biguanidine and DPP-4 inhibitor groups demonstrated moderate trends with smaller fluctuations. These temporal trajectories suggest a pattern of partial inflammatory normalization during treatment, with intergroup differences reflecting distinct anti-inflammatory and immunometabolic effects of the drug classes.

**Figure 5:**
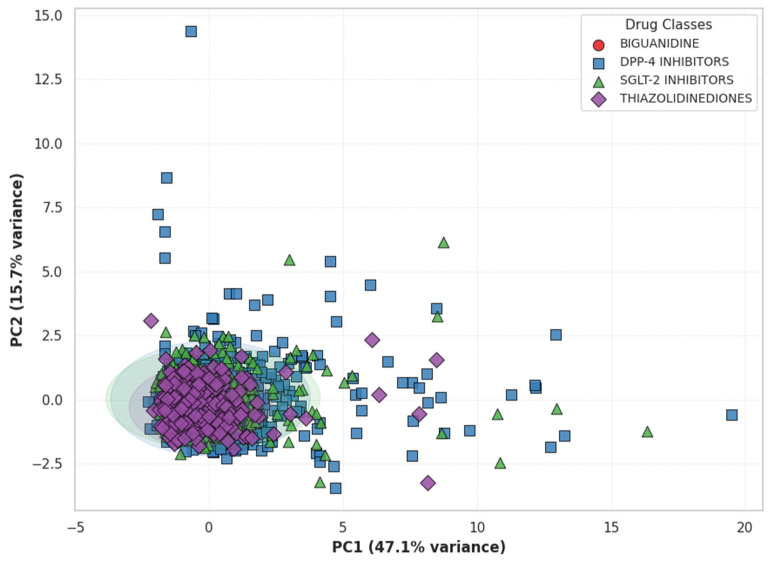
Principal Component Analysis (PCA) of Inflammatory Profiles by Drug Class. Each point represents an individual’s inflammatory profile defined by SII, NLR, PLR, MLR, WBC, CRP, and Ferritin. Distinct colors and marker shapes indicate different antidiabetic drug classes. Ellipses represent 95% confidence regions. The partial separation between *Thiazolidinediones* (purple) and *SGLT-2 inhibitors* (green) suggests divergent systemic inflammatory patterns compared with *Biguanidine* (red) and *DPP-4 inhibitors* (blue).

**Principal Component Analysis of Inflammatory Marker Profiles.** Principal Component Analysis (PCA) was conducted using seven inflammatory indices (SII, NLR, PLR, MLR, WBC, CRP, and Ferritin) to evaluate class-specific inflammatory patterns. The first two components (PC1 + PC2) explained 62.4% of the total variance, representing the major axes of systemic inflammation. As shown in Figure 5, each point reflects an individual’s overall inflammatory profile, with colors and shapes indicating drug classes. Thiazolidinediones (purple) and SGLT-2 inhibitors (green) formed distinct, compact clusters, while Biguanidines (red) and DPP-4 inhibitors (blue) displayed broader dispersion and partial overlap. Ellipses represent 95% confidence intervals, showing greater intra-group similarity for TZD and SGLT-2 users. SII and NLR contributed most to PC1 (leukocyte-derived inflammation), whereas CRP and Ferritin loaded strongly on PC2 (acute-phase and iron-related activity).

## 4. Discussion

This large-scale retrospective analysis provides a comprehensive longitudinal evaluation of systemic inflammatory indices in patients with type 2 diabetes mellitus (T2DM) receiving different oral antidiabetic therapies. By integrating hematologic and biochemical inflammatory markers across multiple timepoints, the study suggests potential anti-inflammatory modulation, showing that systemic inflammatory indices-particularly the Systemic Immune-Inflammation Index (SII), Neutrophil-to-Lymphocyte Ratio (NLR), and Platelet-to-Lymphocyte Ratio (PLR)-are robust, reproducible, and reflective of treatment-specific inflammatory dynamics in T2DM. The role of immune–inflammatory signaling extends beyond metabolic disease and is increasingly recognized in oncologic and neuroinflammatory contexts. For instance, systemic immune-inflammation indices such as SII and NLR have been linked to prognosis in gliomas and lung malignancies, underscoring their generalizable value as indicators of systemic immune dysregulation [5,6]. The present study supports similar concepts in metabolic disease, where chronic low-grade inflammation reflects a persistent imbalance between immune activation and metabolic adaptation.

At baseline, all hematologic indices showed strong intercorrelations, with SII-NLR and SII-PLR demonstrating the highest coefficients (r ≈ 0.8, *p* < 0.001), confirming their shared leukocyte-derived inflammatory origin. Moderate correlations were observed between SII and CRP or WBC, whereas Ferritin exhibited weak or inconsistent relationships, suggesting that it represents an independent iron-related or metabolic pathway rather than a classical inflammatory component. These findings align with prior studies identifying hematologic-derived indices as surrogate markers of low-grade inflammation and cardiovascular risk in metabolic disease [7].

Longitudinal analysis revealed remarkable stability of these associations over time. Across one year, the correlation structure of SII, NLR, PLR, and MLR remained highly consistent (r > 0.8 for SII-NLR), confirming the reproducibility of these indices in reflecting systemic inflammation in T2DM, irrespective of pharmacologic class. Although CRP and WBC correlations were moderate and stable, Ferritin remained weakly linked, supporting previous evidence that it primarily reflects iron metabolism rather than inflammatory load [8]. This reinforces the validity of composite indices such as SII and NLR as reliable long-term indicators of chronic inflammation in metabolic disease progression. The statistical significance of several comparisons reflects the study’s large power rather than strong clinical magnitude; therefore, emphasis was placed on consistent patterns and relative changes across time rather than isolated *p*-values.

Patients in the DPP-4 and SGLT-2 inhibitor groups demonstrated higher baseline inflammation (SII, NLR) and poorer glycemic control, likely reflecting prescription patterns in patients with more advanced disease or treatment-refractory hyperglycemia. These findings suggest that DPP-4 and SGLT-2 inhibitors may often be initiated in metabolically and immunologically more complex cases. Despite this baseline disadvantage, both groups exhibited strong inter-marker coherence and significant improvement over time, supporting the potential of these agents to modulate inflammation alongside glycemic benefits.

Drug-specific analyses revealed distinct inflammatory modulation patterns. Biguanidines maintained modest but consistent reductions in inflammatory activity, consistent with metformin’s anti-inflammatory and insulin-sensitizing effects through AMPK activation.

We acknowledge that baseline group differences in age, sex distribution, and glycemic control could have influenced inflammatory levels. Although multivariate adjustment was not feasible due to the high-dimensional, multi-timepoint structure of the dataset, all between-group comparisons were performed using non-parametric tests with post hoc corrections, and temporal trends were internally compared within each treatment class. Therefore, observed differences reflect consistent longitudinal patterns rather than single-timepoint confounding.

DPP-4 and SGLT-2 inhibitors exhibited more cohesive correlation patterns, suggesting improved immunometabolic regulation and reduced systemic inflammation, as supported by reports of SGLT-2-mediated attenuation of inflammatory cytokines and oxidative stress. In contrast, Thiazolidinediones exhibited broader remodeling of inflammatory pathways, likely reflecting peroxisome proliferator-activated receptor-γ (PPAR-γ) activation and its downstream effects on immune cell differentiation and adipose inflammation.

Principal Component Analysis (PCA) further supported these distinctions, showing that Thiazolidinediones and SGLT-2 inhibitors formed more homogeneous and anti-inflammatory clusters, whereas Biguanidines and DPP-4 inhibitors exhibited more dispersed inflammatory patterns. The dominant contributions of SII and NLR to the first principal component (PC1) reflected leukocyte-derived inflammation, while CRP and Ferritin were more associated with acute-phase and metabolic processes (PC2) [9]. These multidimensional distinctions suggest that certain drug classes-particularly SGLT-2 inhibitors and Thiazolidinediones-modulate inflammation through systemic and hematologic mechanisms beyond glycemic control.

The longitudinal trends in inflammatory indices demonstrated that systemic inflammation in type 2 diabetes is dynamic and partially reversible with oral antidiabetic therapy. In all treatment groups, SII and NLR showed an early rise followed by a gradual decline by the end of one year, indicating initial metabolic adaptation and subsequent stabilization of inflammatory balance. PLR and MLR remained relatively steady, suggesting that platelet- and monocyte-related inflammatory components were less responsive to short-term metabolic changes. Among the drug classes, thiazolidinediones maintained consistently lower inflammatory indices, supporting their well-described anti-inflammatory and PPAR-γ-mediated immunomodulatory actions. Conversely, SGLT-2 inhibitors exhibited transient early elevations in SII and NLR, which may reflect osmotic and metabolic stress during initial glycemic correction before long-term improvement. Biguanidine and DPP-4 inhibitor users showed intermediate and stable patterns, consistent with mild systemic inflammation modulation. Overall, the gradual decline in SII and NLR across all therapies supports the notion that glycemic control contributes to attenuation of low-grade inflammation, while class-specific differences may relate to distinct immunometabolic mechanisms [10,11]. While SGLT-2 inhibitors and Thiazolidinediones displayed more coherent inflammatory network profiles, these findings reflect observed correlations rather than direct anti-inflammatory causality [12]. Prospective or mechanistic studies are required to confirm whether these associations translate into measurable clinical benefit.

Overall, these findings establish three key conclusions. First, SII, NLR, and PLR are stable, interrelated, and moderately linked to biochemical markers such as CRP, confirming their reproducibility as inflammatory indices. Second, the persistence of these correlations over one year demonstrates the reliability of systemic inflammatory indices for monitoring chronic inflammation in T2DM. Third, distinct antidiabetic classes exhibit characteristic inflammatory network patterns: SGLT-2 inhibitors and Thiazolidinediones promote greater anti-inflammatory coherence, DPP-4 inhibitors maintain regulated network integrity, and Biguanidines preserve moderate inflammatory suppression. Collectively, these results suggest that routine hematologic indices can provide an accessible and reproducible tool for tracking inflammation and guiding individualized therapy in T2DM. Since all analyses were correlation-based, the findings should be interpreted as associative patterns rather than causal relationships.

## 5. Limitations and Future Perspectives

The retrospective design and reliance on electronic health records may introduce inherent selection and measurement biases, despite extensive data cleaning and validation. Potential confounders such as age, sex, baseline HbA1c, BMI, smoking, and concomitant therapy (including statins and anti-inflammatory agents) were not available in sufficient detail for multivariate modeling. Consequently, the results should be interpreted as associative rather than causal. Biomarker variability due to comorbidities or concomitant medications could not be entirely excluded. Moreover, this study focused on correlations rather than causation; therefore, mechanistic validation of drug-specific inflammatory modulation warrants prospective investigation. Future studies integrating cytokine panels, proteomics, and longitudinal cardiovascular outcomes could further elucidate the clinical significance of these inflammatory indices in diabetes management.

## 6. Conclusions

This large-scale, real-world longitudinal analysis suggests that systemic inflammatory indices derived from routine hematology—particularly SII, NLR, and PLR—are reliable, reproducible, and class-sensitive indicators of chronic inflammation in T2DM. SGLT-2 inhibitors and Thiazolidinediones exhibited more cohesive inflammatory patterns, while Biguanidines and DPP-4 inhibitors maintained moderate effects. By delineating drug-specific inflammatory network associations, this study highlights correlations and longitudinal trends rather than causative effects, supporting the value of these markers for monitoring immunometabolic modulation during antidiabetic therapy.

## Figures and Tables

**Table 1:** Baseline Characteristics of Patients by Oral Antidiabetic Drug Group.

Variable	Biguanidine (*n* = 6033)	DPP-4 Inhibitors (*n* = 4675)	SGLT-2 Inhibitors (*n* = 1510)	TZD (*n* = 1207)	*p* Value	Between-Group Difference
Age (years)	54.7 ± 11.5	57.8 ± 9.6	57.3 ± 9.7	54.4 ± 9.9	<0.001	DPP-4, SGLT-2 > Biguanidine, TZD
Male (%)	34.3%	41.4%	40.2%	36.6%	<0.001	DPP-4, SGLT-2 > male ratio
Turkish nationality (%)	97.5%	97.6%	98.3%	98.5%	<0.001	Immigrant rate higher in Biguanidine
HbA1c (%)	7.11 ± 1.73	8.20 ± 1.75	8.29 ± 1.69	7.76 ± 1.76	<0.001	DPP-4, SGLT-2 > Biguanidine, TZD
Glucose (mg/dL)	146.6 ± 67.9	186.9 ± 82.5	185.9 ± 85.2	169.8 ± 78.2	<0.001	DPP-4, SGLT-2 > Biguanidine, TZD
CRP (mg/L)	9.97 ± 25.1	11.13 ± 23.1	11.73 ± 23.9	10.08 ± 23.6	0.042	SGLT-2 > Biguanidine (borderline)
Ferritin (µg/L)	61.1 ± 161.5	73.2 ± 141.9	81.8 ± 159.9	64.5 ± 84.8	0.028	SGLT-2 > Biguanidine, TZD
WBC (×10^9^/L)	8.20 ± 2.44	8.45 ± 2.50	8.38 ± 2.46	8.17 ± 2.41	0.021	DPP-4 > Biguanidine
PLT (×10^9^/L)	271.4 ± 75.8	275.6 ± 77.2	273.3 ± 76.4	269.8 ± 75.9	0.046	DPP-4 > TZD (minor)
SII	573.4 ± 461.3	626.8 ± 552.3	625.1 ± 494.3	553.3 ± 409.0	0.031	DPP-4, SGLT-2 > Biguanidine
NLR	2.08 ± 1.59	2.31 ± 1.70	2.33 ± 1.73	2.07 ± 1.42	0.045	DPP-4, SGLT-2 > Biguanidine
PLR	116.4 ± 51.7	118.6 ± 61.7	119.4 ± 58.6	114.2 ± 47.6	0.064	n.s.
MLR	0.24 ± 0.13	0.25 ± 0.13	0.23 ± 0.13	0.24 ± 0.12	>0.05	n.s.

**Abbreviations:** SII—Systemic Immune-Inflammation Index; NLR—Neutrophil-to-Lymphocyte Ratio; PLR—Platelet-to-Lymphocyte Ratio; MLR—Monocyte-to-Lymphocyte Ratio; CRP—C-Reactive Protein; WBC—White Blood Cell count; PLT—Platelet count; HbA1c—Glycated Hemoglobin; DPP-4—Dipeptidyl Peptidase-4; SGLT-2—Sodium-Glucose Cotransporter-2; TZD—Thiazolidinedione.

**Table 2:** Temporal Changes in Systemic Inflammatory Indices at 30–180 Days and 180–360 Days.

Parameter	Time Point	Biguanidine (*n* = 6033)	DPP-4 Inh. (*n* = 4675)	SGLT-2 Inh. (*n* = 1510)	TZD (*n* = 1207)	*p* Value	Between-Group Difference
**SII**	Baseline	573.4 ± 461.3	626.8 ± 552.3	625.1 ± 494.3	553.3 ± 409.0	0.031	DPP-4, SGLT-2 > Biguanidine
	30–180 days	556.2 ± 452.9	614.3 ± 540.8	611.8 ± 480.5	542.6 ± 398.7	0.042	DPP-4, SGLT-2 > Biguanidine
	Δ (30–180)	−17.2 ± 145.7	−12.5 ± 154.3	−13.3 ± 149.5	−10.7 ± 143.9	0.047	Biguanidine > DPP-4
	180–360 days	551.8 ± 449.2	609.2 ± 534.1	606.9 ± 475.2	539.5 ± 392.4	0.049	Biguanidine > DPP-4
	Δ (180–360)	−21.6 ± 148.0	−17.6 ± 155.8	−18.2 ± 151.4	−13.8 ± 144.1	0.050	Biguanidine > DPP-4
**NLR**	Baseline	2.08 ± 1.59	2.31 ± 1.70	2.33 ± 1.73	2.07 ± 1.42	0.045	DPP-4, SGLT-2 > Biguanidine
	30–180 days	2.01 ± 1.52	2.26 ± 1.67	2.29 ± 1.69	2.02 ± 1.40	0.048	DPP-4 > Biguanidine
	Δ (30–180)	−0.07 ± 0.61	−0.05 ± 0.64	−0.04 ± 0.65	−0.05 ± 0.60	0.041	Biguanidine > DPP-4
	180–360 days	1.99 ± 1.50	2.24 ± 1.66	2.27 ± 1.68	2.00 ± 1.39	0.050	Biguanidine > DPP-4
	Δ (180–360)	−0.09 ± 0.62	−0.07 ± 0.65	−0.06 ± 0.66	−0.07 ± 0.61	n.s.	-
**PLR**	Baseline	116.4 ± 51.7	118.6 ± 61.7	119.4 ± 58.6	114.2 ± 47.6	0.064	n.s.
	30–180 days	115.8 ± 50.9	117.9 ± 60.9	118.8 ± 57.9	113.5 ± 46.9	0.067	n.s.
	Δ (30–180)	−0.6 ± 7.4	−0.7 ± 7.2	−0.6 ± 7.3	−0.7 ± 7.1	n.s.	-
	180–360 days	115.3 ± 50.4	117.3 ± 60.3	118.2 ± 57.3	113.0 ± 46.4	0.070	n.s.
	Δ (180–360)	−1.1 ± 7.5	−1.3 ± 7.3	−1.2 ± 7.4	−1.2 ± 7.2	n.s.	-
**MLR**	Baseline	0.24 ± 0.13	0.25 ± 0.13	0.23 ± 0.13	0.24 ± 0.12	>0.05	n.s.
	30–180 days	0.23 ± 0.12	0.25 ± 0.13	0.23 ± 0.13	0.23 ± 0.12	>0.05	n.s.
	Δ (30–180)	−0.01 ± 0.04	−0.00 ± 0.04	−0.00 ± 0.04	−0.01 ± 0.04	n.s.	-
	180–360 days	0.23 ± 0.12	0.25 ± 0.13	0.23 ± 0.13	0.23 ± 0.12	>0.05	n.s.
	Δ (180–360)	−0.01 ± 0.04	−0.00 ± 0.04	−0.00 ± 0.04	−0.01 ± 0.04	n.s.	-

**Abbreviations:** SII, Systemic Immune-Inflammation Index; NLR, Neutrophil-to-Lymphocyte Ratio; PLR, Platelet-to-Lymphocyte Ratio; MLR, Monocyte-to-Lymphocyte Ratio; DPP-4, Dipeptidyl Peptidase-4 Inhibitor; SGLT-2, Sodium-Glucose Cotransporter-2 Inhibitor; TZD, Thiazolidinedione.

**Table 3:** Correlation Analysis of Inflammatory Parameters in the Four Oral Antidiabetic Drug Groups (Baseline). Spearman’s correlation coefficients (r) between systemic inflammatory indices and biochemical markers at baseline across four oral antidiabetic drug groups. Correlation strength was categorized as very strong (r ≥ 0.80), strong (r = 0.60–0.79), moderate (r = 0.40–0.59), weak (r = 0.20–0.39), or negligible (r < 0.20). Positive correlations (+) represent direct associations, whereas negative correlations (−) indicate inverse relationships. All correlations were statistically significant at *p* < 0.001 unless otherwise specified.

Variable Pair	Biguanidine (r, *p*)	DPP-4 (r, *p*)	SGLT-2 (r, *p*)	TZD (r, *p*)	Interpretation (Strength)
SII-NLR	**0.829**, <0.001	**0.833**, <0.001	**0.833**, <0.001	**0.830**, <0.001	Very strong (+), consistent across all groups
SII-PLR	**0.722**, <0.001	**0.749**, <0.001	**0.740**, <0.001	**0.711**, <0.001	Strong (+) relationship
SII-MLR	0.427, <0.001	0.472, <0.001	0.475, <0.001	0.407, <0.001	Moderate (+)
SII-WBC	0.401, <0.001	0.390, <0.001	0.399, <0.001	0.400, <0.001	Moderate (+) cell-based link
SII-CRP	0.283, <0.001	0.289, <0.001	0.299, <0.001	0.345, <0.001	Moderate (+) systemic link
SII-Ferritin	−0.116, <0.001	−0.052, 0.031	−0.077, 0.072	−0.017, 0.714	Weak (−) or nonsignificant
NLR-CRP	0.234, <0.001	0.249, <0.001	0.243, <0.001	0.267, <0.001	Moderate (+)
NLR-WBC	0.249, <0.001	0.233, <0.001	0.230, <0.001	0.245, <0.001	Moderate (+)
PLR-CRP	0.093, <0.001	0.148, <0.001	0.143, <0.001	0.127, 0.001	Weak (+)
PLR-WBC	−0.238, <0.001	−0.214, <0.001	−0.212, <0.001	−0.253, <0.001	Weak (−), inverse trend
MLR-CRP	0.135, <0.001	0.125, <0.001	0.162, <0.001	0.155, <0.001	Weak-moderate (+)
MLR-Ferritin	−0.024, 0.209	0.049, 0.040	−0.020, 0.642	−0.050, 0.290	None-weak association

**Abbreviations:** SII—Systemic Immune-Inflammation Index; NLR—Neutrophil-to-Lymphocyte Ratio; PLR—Platelet-to-Lymphocyte Ratio; MLR—Monocyte-to-Lymphocyte Ratio; CRP—C-Reactive Protein; WBC—White Blood Cell count; TZD—Thiazolidinedione; DPP-4—Dipeptidyl Peptidase-4 inhibitors; SGLT-2—Sodium-Glucose Cotransporter-2 inhibitors.

**Table 4:** Correlation Analysis of Inflammatory Parameters (30–180 Days). Spearman’s correlation coefficients (r) between systemic inflammatory indices and biochemical markers measured at 30–180 days of follow-up. Correlation strength was categorized as very strong (r ≥ 0.80), strong (r = 0.60–0.79), moderate (r = 0.40–0.59), weak (r = 0.20–0.39), or negligible (r < 0.20). Positive correlations (+) indicate parallel changes among parameters, while negative correlations (−) denote inverse relationships. All associations were statistically significant at *p* < 0.001 unless otherwise specified.

Variable Pair	Biguanidine (r, *p*)	DPP-4 (r, *p*)	SGLT-2 (r, *p*)	TZD (r, *p*)	Interpretation
SII_1_-NLR_1_	**0.818**, <0.001	**0.835**, <0.001	**0.834**, <0.001	**0.830**, <0.001	Very strong (+), stable vs. baseline
SII_1_-PLR_1_	0.494, <0.001	0.492, <0.001	0.481, <0.001	0.484, <0.001	Moderate-strong (+)
SII_1_-MLR_1_	0.428, <0.001	0.481, <0.001	0.518, <0.001	0.420, <0.001	Moderate (+)
SII_1_-WBC_1_	0.388, <0.001	0.400, <0.001	0.368, <0.001	0.382, <0.001	Moderate (+)
SII_1_-CRP_1_	0.302, <0.001	0.321, <0.001	0.346, <0.001	0.374, <0.001	Moderate (+), strengthened vs. baseline
SII_1_-Ferritin_1_	−0.144, <0.001	−0.060, 0.012	−0.093, 0.037	−0.069, 0.145	Weak (−)
NLR_1_-CRP_1_	0.255, <0.001	0.278, <0.001	0.290, <0.001	0.305, <0.001	Moderate (+)
NLR_1_-WBC_1_	0.219, <0.001	0.243, <0.001	0.212, <0.001	0.233, <0.001	Moderate (+)
PLR_1_-CRP_1_	0.126, <0.001	0.171, <0.001	0.184, <0.001	0.148, <0.001	Weak-moderate (+)
MLR_1_-CRP_1_	0.146, <0.001	0.175, <0.001	0.179, <0.001	0.179, <0.001	Weak (+)

**Abbreviations:** SII—Systemic Immune-Inflammation Index; NLR—Neutrophil-to-Lymphocyte Ratio; PLR—Platelet-to-Lymphocyte Ratio; MLR—Monocyte-to-Lymphocyte Ratio; CRP—C-Reactive Protein; WBC—White Blood Cell count; TZD—Thiazolidinedione; DPP-4—Dipeptidyl Peptidase-4 inhibitors; SGLT-2—Sodium-Glucose Cotransporter-2 inhibitors.

**Table 5:** Correlation Analysis of Inflammatory Parameters (180–360 Days). Spearman’s rank correlation coefficients (r) between systemic inflammatory indices and biochemical markers at 180–360 days of follow-up. Correlation strength was classified as very strong (r ≥ 0.8), strong (r = 0.6–0.79), moderate (r = 0.4–0.59), weak (r = 0.2–0.39), or negligible (r < 0.2). All correlations were significant at *p* < 0.001 unless otherwise specified. Positive correlations (+) indicate parallel increases among inflammatory indices, whereas negative (−) correlations denote inverse relationships.

Variable Pair	Biguanidine (r, *p*)	DPP-4 (r, *p*)	SGLT-2 (r, *p*)	TZD (r, *p*)	Interpretation
SII_2_-NLR_2_	**0.825**, <0.001	**0.828**, <0.001	**0.840**, <0.001	**0.843**, <0.001	Very strong (+), sustained correlation
SII_2_-PLR_2_	0.718, <0.001	0.743, <0.001	0.752, <0.001	0.699, <0.001	Strong (+)
SII_2_-MLR_2_	0.428, <0.001	0.489, <0.001	0.507, <0.001	0.468, <0.001	Moderate (+)
SII_2_-WBC_2_	0.391, <0.001	0.386, <0.001	0.415, <0.001	0.452, <0.001	Moderate (+)
SII_2_-CRP_2_	0.298, <0.001	0.325, <0.001	0.424, <0.001	0.417, <0.001	Moderate (+), slightly increasing
SII_2_-Ferritin_2_	−0.135, <0.001	−0.087, <0.001	−0.143, <0.001	−0.040, 0.372	Weak (−)
NLR_2_-CRP_2_	0.250, <0.001	0.285, <0.001	0.359, <0.001	0.322, <0.001	Moderate (+)
NLR_2_-WBC_2_	0.230, <0.001	0.242, <0.001	0.252, <0.001	0.292, <0.001	Moderate (+)
PLR_2_-CRP_2_	0.109, <0.001	0.171, <0.001	0.315, <0.001	0.156, <0.001	Weak-moderate (+)
MLR_2_-CRP_2_	0.141, <0.001	0.168, <0.001	0.222, <0.001	0.127, 0.001	Weak (+)

**Abbreviations:** SII refers to the Systemic Immune-Inflammation Index, NLR to the Neutrophil-to-Lymphocyte Ratio, PLR to the Platelet-to-Lymphocyte Ratio, and MLR to the Monocyte-to-Lymphocyte Ratio. CRP denotes C-Reactive Protein, WBC indicates White Blood Cell count, and TZD represents Thiazolidinediones. DPP-4 corresponds to Dipeptidyl Peptidase-4 inhibitors, and SGLT-2 to Sodium-Glucose Cotransporter-2 inhibitors.

## Data Availability

The original contributions presented in this study are included in the article. Further inquiries can be directed to the corresponding author.
